# A geospatial risk analysis graphical user interface for identifying hazardous chemical emission sources

**DOI:** 10.7717/peerj.14664

**Published:** 2023-01-18

**Authors:** Hongfei Hou, Huiying Ren, Patrick Royer, Xiao-Ying Yu

**Affiliations:** 1 Energy and Environment Directorate, Pacific Northwest National Laboratory, Richland, WA, United States of America; 2 Materials Science and Technology Division, Oak Ridge National Laboratory, Oak Ridge, TN, United States of America

**Keywords:** HYSPLIT, PSDF, Open source, Graphical user interface, Geospatial risk, Chemical hazard assessment, Software engineering, Computer science

## Abstract

**Background:**

Performing back trajectory and forward trajectory using the Hybrid Single-Particle Lagrangian Integrated Trajectory Model (HYSPLIT) is a reliable approach for assessing particle transport after release among mid-field atmospheric models. HYSPLIT has an externally facing online interface that allows non-expert users to run the model trajectories without requiring extensive training or programming. However, the existing HYSPLIT interface is limited if simulations have a large amount of meteorological data and timesteps that are not coincident. The objective of this study is to design and develop a more robust tool to rapidly evaluate hazard transport conditions and to perform risk analysis, while still maintaining an intuitive and user-friendly interface.

**Methods:**

HYSPLIT calculates forward and backward trajectories of particles based on wind speed, wind direction, and the corresponding location, timestamp, and Pasquill stability classes of the regions of the atmosphere in terms of the wind speed, the amount of solar radiation, and the fractional cloud cover. The computed particle transport trajectories, combined with the online Proton Transfer Reaction-Mass Spectrometry (PTR-MS) data (https://figshare.com/articles/dataset/ARL_Data_from_PROS_station_at_Hanford_site/19993964), can be used to identify and quantify the sources and affected area of the hazardous chemicals’ emission using the potential source distribution function (PSDF). PSDF is an improved statistical function based on the well-known potential source contribution function (PSCF) in establishing the air pollutant source and receptor relationship. Performing this analysis requires a range of meteorological and pollutant concentration measurements to be statistically meaningful. The existing HYSPLIT graphical user interface (GUI) does not easily permit computations of trajectories of a dataset of meteorological data in high temporal frequency. To improve the performance of HYSPLIT computations from a large dataset and enhance risk analysis of the accidental release of material at risk, a geospatial risk analysis tool (GRAT-GUI) is created to allow large data sets to be processed instantaneously and to provide ease of visualization.

**Results:**

The GRAT-GUI is a native desktop-based application and can be run in any Windows 10 system without any internet access requirements, thus providing a secure way to process large meteorological datasets even on a standalone computer. GRAT-GUI has features to import, integrate, and convert meteorological data with various formats for hazardous chemical emission source identification and risk analysis as a self-explanatory user interface. The tool is available at https://figshare.com/articles/software/GRAT/19426742.

## Introduction

Emissions of hazardous chemicals can lead to negative effects on health, including birth defects, nervous systems problems, and cancer (EPA, 2022). Heightened levels of risk to the health of human beings and wildlife are observed in regions where climate patterns are more conducive to increased particle mobility and transport, allowing hazardous chemicals to vaporize more easily into the air (Pamela & Lema, 2015; Jung & Qassimi, 2022). Environmental regulatory requirements frequently call for risk assessments to balance the tradeoffs between economic output and environmental protection.

Identifying the potential sources and affected areas due to emissions of hazardous chemicals plays a critical role in risk assessment because it supports decision-making and protects workers and communities from being incidentally exposed to chemical hazards within the identified areas (TALA, 2001). Real-time volatile organic compound (VOC) measurements, such as those from modern analytical instruments like proton transfer mass spectrometer (PTR-MS), coupled with long-term meteorological data (Zhou et al., 2022) are necessary to understand the pollutant concentrations at the emission point. Atmospheric dispersion models are used to perform trajectory simulations of particles formed as a result of VOCs emitted into the air (Stockie, 2011). Lagrangian models are often the physical base of trajectory simulations. Among known models, HYbrid Single-Particle Lagrangian Integrated Trajectory, namely HYSPLIT, is well established. It was first developed by the National Oceanic and Atmospheric Administration (NOAA) Air Resources Laboratory and the Australian Bureau of Meteorology Research Center. To trace the particle emission source and receptor relationship, statistical functions are employed. Specifically, the Potential Source Contribution Function (PSCF) is used to analyze the variation of potential sources and transports of chemical hazards (Ashbaugh, Malm & Sadeh, 1985; Cheng et al., 1993; Peng, Choi & Wong, 2007; Ren et al., 2019; Yu, 2013; Zeng & Hopke, 1989). It is worth noting that PSCF not only can identify but also quantify the source areas by incorporating the Gaussian process regression (Williams & Rasmussen, 1995). Most recently, an improved potential source distribution function (PSDF) is introduced, making the statistical approach more powerful (Kim, Kim & Wee, 2022).

PTR-MS data is used to identify and determine the concentration of hazardous chemicals coincident with meteorological data. The nature of PTR-MS data, specifically its high temporal resolution and high detection sensitivity of VOCs, makes it a suitable technique for real-time monitoring of multiple VOC emissions (Kaklamanos, Aprea & Theodoridis, 2016). In this case study, we analyze PTR-MS from tank farms in the Hanford site where meteorological measurements are routinely collected. The Hanford cleanup site has 149 single-shell tanks and 28 double-shell tanks grouped into “tank farms” to store radioactive waste. In order to maintain the atmospheric equilibrium between tanks and surroundings, breathers are used to vent gasses, which uses filters to neutralize the harmful effects of gasses. PTR-MS collection was established to monitor air quality and potential hazards for the nearby workforce in the vicinity of tank breathers. PTR-MS data is usually collected at 1 Hz from the Hanford site, and they can be resampled with longer time intervals for meaningful analysis of trajectory calculations using physics-based models.

Trajectories data is computed from meteorological data using the HYSPLIT model (Draxler et al., 2020; Stein et al., 2015). The HYSPLIT model can calculate two types of trajectories. Forward trajectories are used for predicting where the air particle will travel, while backward trajectories are used for identifying the sources of pollutant emissions. Currently, there are four versions of HYSPLIT. The first version was developed in 1982, and the last one in 1998. HYSPLIT comes with an online version and a local version of GUI. HYSPLIT GUI provides functionality to enable users to enter their meteorological data including wind speed, wind direction, time stamp, and stabilities. Before computing trajectories, HYPLIT will convert the meteorological data into the Air Resources Laboratory (ARL) data format, which provides a fixed-length record for each meteorological variable (NOAA, 2022). For example, users can fill in up to six data entries for each conversion, which takes about 4 min. Thus, for a 10-year meteorological data set with 15-minute resolution, it will take 3,894 h for data entry, which will be too long to be practical. Therefore, the available HYSPLIT-GUI makes it difficult to handle a large number of data containing continuous meteorological measurements of days or weeks.

We develop a GRAT to increase efficiency for the computational process. It enables users to perform a batch process of meteorological data reduce time involved in required data conversion, especially for large datasets. GRAT is a comprehensive toolset to generate inputs to run the PSDF model, the latter was recently developed and updated from PSCF (Daehyun, 2022). GRAT can directly load data in the comma-separated values (CSV) format with minimum user interaction and perform conversion of the entire file in one operation. This feature will prevent inaccurate data entry caused by human error and significantly reduce the workload since users can directly use the data retrieved from the measurement site’s database without manual entry. The latter is required when using the HYSPLIT GUI. GRAT is also designed to compute the converted ARL file for trajectory calculations and correspond to the resampled PTR-MS measurements at different time intervals for meaningful interpretation. Both types of observed data will serve as the input of PSDF to determine the source and influenced area due to emissions of hazardous chemicals.

## Materials & Methods

### Data sources and description

**Figure 1:**
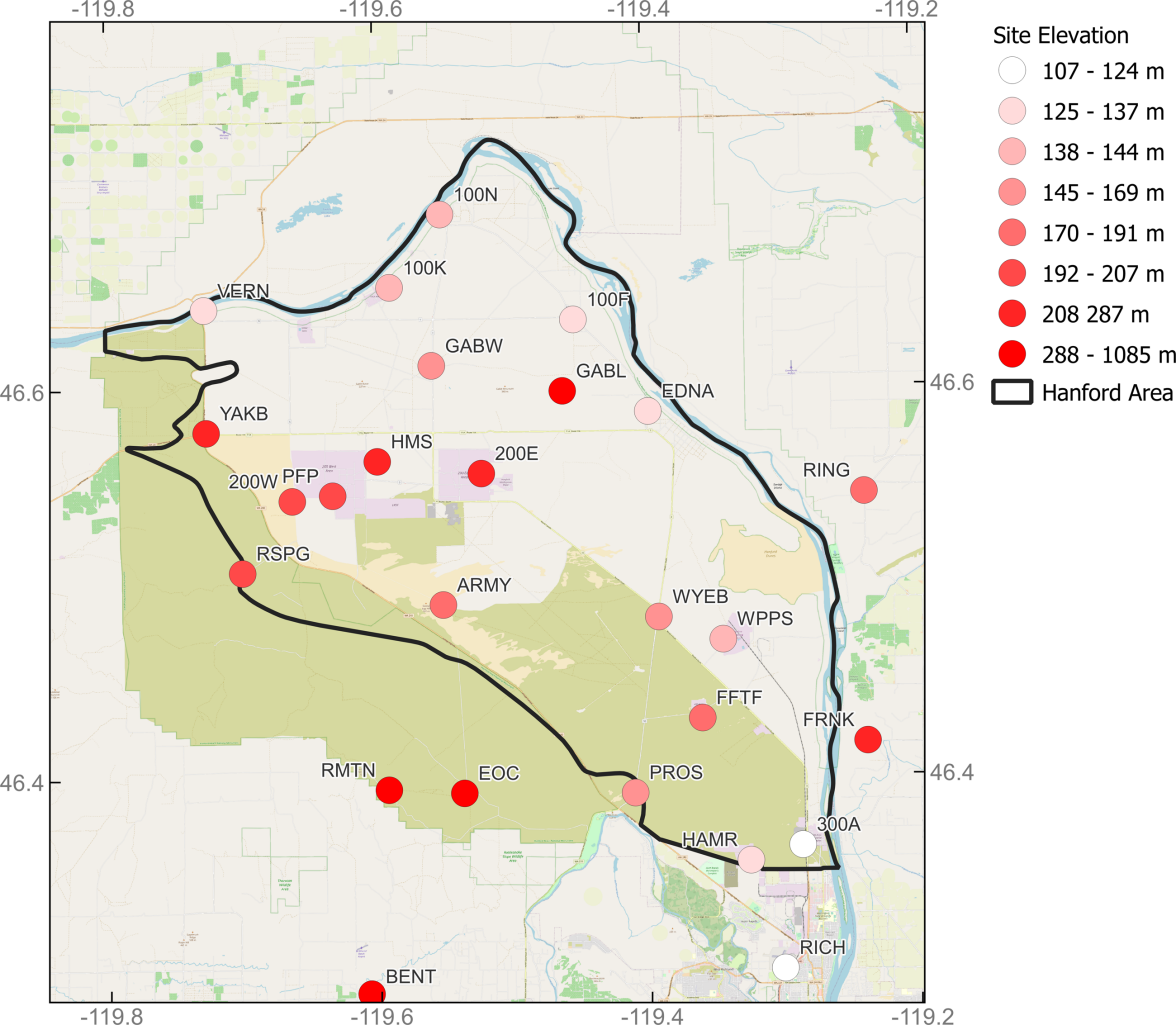
The map of meteorological monitoring stations in the Hanford site (Hanford, 2022) created using the Free and Open Source QGIS. The maximum sea level of the stations is 330 m, and the minimum is 110 m.

The data package used in this study is retrieved from the meteorological database of the Hanford site, which consists of 30 monitoring stations. Hanford is located in the Benton County of Washington state, bordered on the southeast of a metropolitan area composed of Richland, Kennewick, and Pasco. The meteorological and PTR-MS data were resampled from 1 Hz real-time data into datasets of an interval of 15-minute and 1-hour, respectively. Trajectories were computed using the back-trajectory option *via* GRAT based on the meteorological input. The developed application is designed to use the following three different data types:

1. Meteorological data at the Hanford site. These measurements are acquired at over thirty local monitoring stations (Fig. 1), which are operated by the Mission Support Alliance for the US Department of Energy (DOE). Measurements are recorded at varying elevations including the 125-m (400-ft) and three 60-m (200-ft) towers (United States Department of Energy, 2022). Base data includes wind speed, wind direction, and the corresponding recording time (https://www.hanford.gov/page.cfm/HMS). The wind speed is recorded using miles per hour (mph). Wind direction is recorded in standard azimuth measured in degrees clockwise from north on an azimuth circle, such that 270 degrees corresponds to prevailing winds from the west and 180 degrees corresponds to prevailing winds from the south. Data used in this study is from March 1, 2010, to December 31, 2019 (https://www.mdpi.com/article/10.3390/atmos13010136/s1).
2. PTR-MS VOC measurements. The online VOC measurements are acquired by an ultra-sensitive real-time trace gas analyzer, namely PTR-MS Trace VOC analyzer (IONICON Analytik) installed in a mobile laboratory (WRPS, 2019; Majchrzak et al., 2018). The measurements include dozens of VOCs deemed as chemicals of potential concern (*e.g.*, formaldehyde, styrene) (Burgeson et al., 2004). Additional measurements from the mobile platform are available, including ammonia and carbon dioxide. At the Hanford site, there are 19 tank farms where direct-measurement instruments are placed. Data is reported at a 1-minute interval resampled from the original 1 Hz rate. Data used in this study were acquired between December 9, 2016, to September 2, 2017 (https://doi.org/10.5281/zenodo.7369298). QA/QCed data were used in the illustration of the software applications.
3. PSDF calculations. PSDF calculations were done using the back trajectory results from the HYSPLIT model and PTR-MS data. The PSDF results are presented in two formats, namely, a CSV file and a plot. The latitude range is from 43 to 49, and the longitude is from −123 to −117. The dimension of the grid of PSDF in GRAT-GUI is 60 by 60. Thus, each grid cell has a width of 0.1 unit of longitude and a height of 0.1 unit of latitude. The whole geographic region defined by the provided latitude and longitude range is divided into an array of grid cells (a total of 3,600 cells). The CSV file and plot contain calculated results, where larger values indicate a higher likelihood to be the sources for the associated grid cells.

Meteorological data files are in CSV format, which will be processed by GRAT to generate the trajectory data files. The converted ARL format file for the site “PROS” is included in the software installation package and can be accessed in the “Results\ARLData” sub-directory under the installation path. The software-generated “List.xlsx” file contains the trajectory files corresponding to different time stamps and the corresponding PTR-MS data required to run PSDF. The 2D PSDF result plot is saved as a portable network graphics file for visualization on the map.

### Graphical user interface

The GRAT-GUI interface was created as an application compatible with Windows operating system *via* the Microsoft.NET framework. It was developed using the C# programing language and its supplementary packages. It is a standalone desktop-based application that can be run on a local machine which should be installed with a Windows 10 or higher operating system.

**Figure 2:**
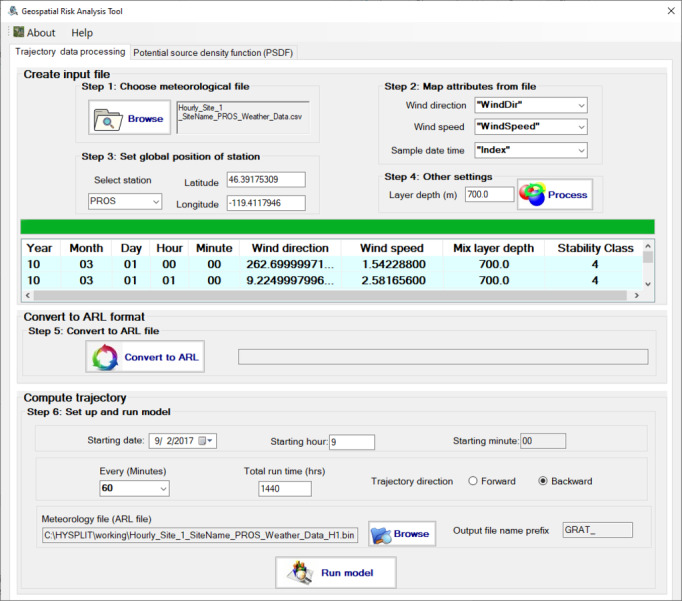
The “Trajectory data processing” tab allows trajectories to be computed from selected meteorological data with a range of options.

The GRAT-GUI interface consists of two tabs. The first one contains all controls to import meteorological data, convert it into the ARL format, then compute trajectories. The “Trajectory data processing” tab shown in Fig. 2 is organized based on the following steps to compute trajectories from meteorological data files.

The GRAT-GUI provides a simple and intuitive user interface. The controls are grouped into different tasks based on the desired purpose. There are three main task blocks in the “Trajectory data processing” tab. The first one is to create the input file for data format conversion. The mapping features enable the GRAT-GUI to support various layouts of the CSV files as input for meteorological measurements from different sites that have their own designated data formats. The interactive table shows the content of the created file and permits users to perform data verification. The second tab is to convert meteorological data into the ARL format.

To process data *via* GRAT_GUI, the first step is to select the proper meteorological data of interest. The CSV format is set as default, including wind speed, wind direction, and timestamp columns. The timestamp column should contain information about the year, month, day, and hour of the measurements. After selecting the meteorological data file, users can use GRAT-GUI to map data included in the selected specific time frame with regard to wind direction, wind speed, and time, respectively. The third and fourth steps are used to input and select information on the longitude and latitude of the monitoring station and the boundary layer depth, respectively. Both are needed in the HYSPLIT model to properly calculate the particle transport path. These data are input in the compatible data format for HYSPLIT calculations. Namely, they are converted into the ARL format using the executable “stn2arl.exe” included in the installed HYSPLIT software. The final step is to set up and run the model to compute trajectories. To identify emission sources and influenced areas, the back trajectory option should be selected. To predict the potentially impacted area from an emission source, the forward trajectory option should be chosen. The GRAT-GUI uses the executable hyts_std.exe from HYSPLIT to create trajectory files.

**Figure 3:**
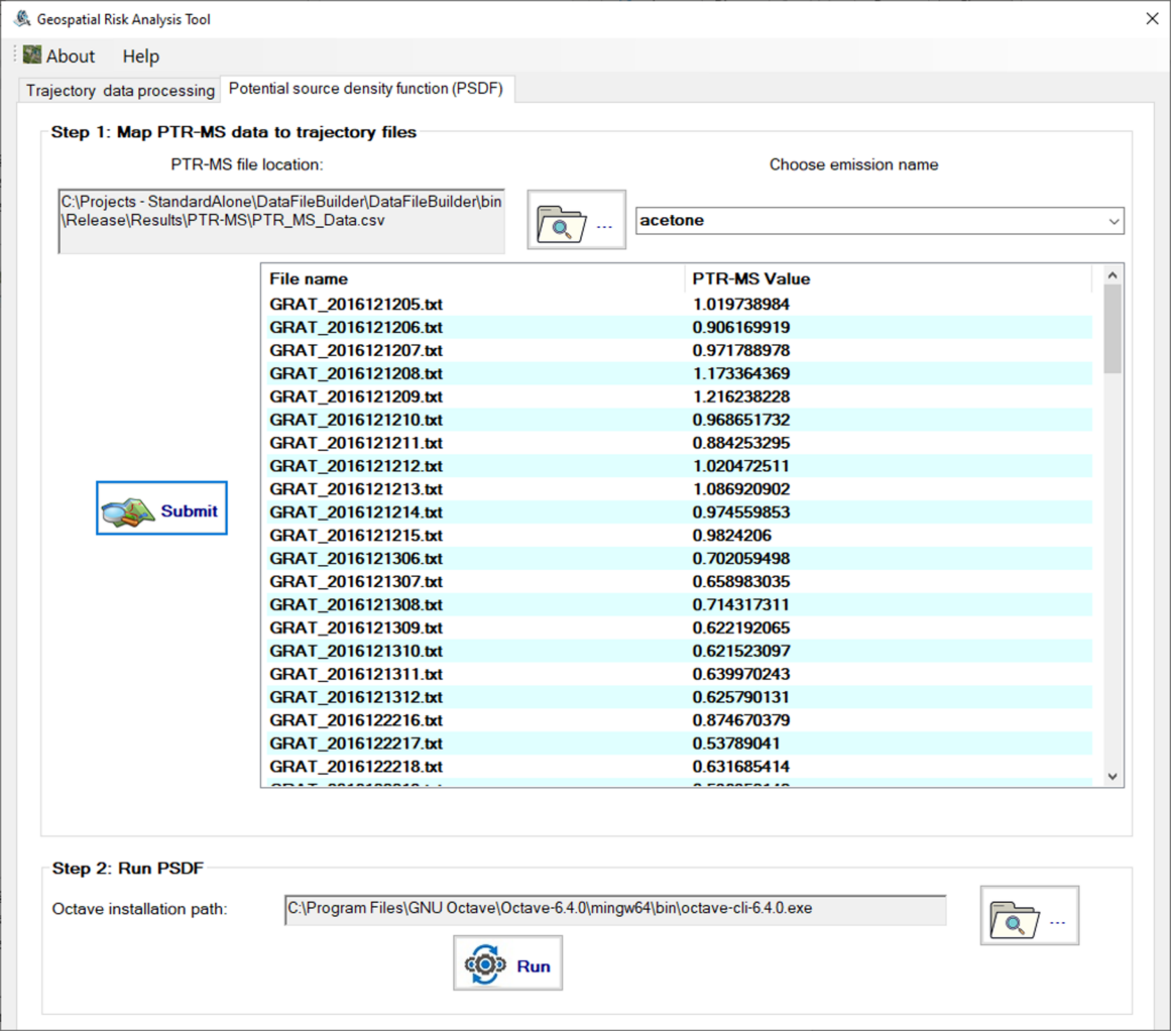
The “Potential source distribution function (PSDF)” tab provides features to choose chemicals, run the PSDF, and show the trajectory results of chemicals measured by PTR-MS on the map.

The “PSDF” tab shown in Fig. 3 allows users to choose the hazardous chemical of interest and generate the required input to run PSDF. After running the execution file of PSDF, the trajectory result plots will be saved into the “Results” directory under the installation folder.

### Availability

The GRAT-GUI can be run under Windows 10 or higher which should have HYSPLIT, and Octave installed. The local computer should have at least 1 GHz (GHz) processor, at least 4 GB (GB) of RAM (8 GB recommended), and at least 16 GB (GB) of available space on the hard disk. HYSPLIT should be installed into the “C:\HYSPLIT” directory. To support a large meteorological dataset, the computer should have at least 3 GB of available RAM. Octave is used to run the PSDF code. The installer of GRAT-GUI is available at https://figshare.com/articles/software/GRAT/19426742 and the data package is available under the “results\MetData” and “results\PTR-MS” subfolder of the installed GRAT-GUI.

## Results and Discussion

**Figure 4:**
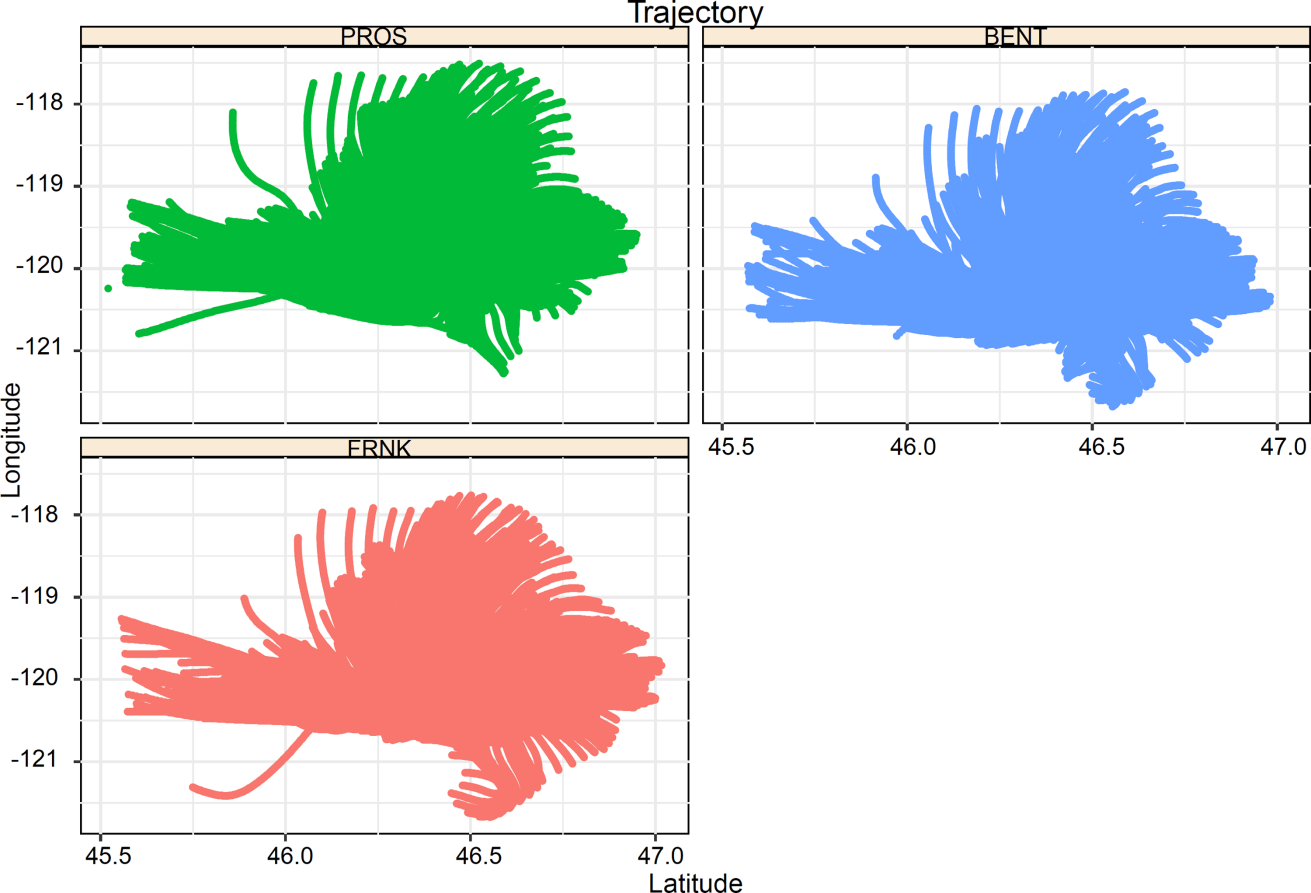
Plots created using a standalone R script showing backward trajectories in the GRAT-GUI. The study case used data from three monitoring stations at the Hanford site: PROS, BENT, and FRNK, with latitude and longitude of (46.39175, −119.41179), (46.28989, −119.60743), and (46.41693, −119.2375), respectively.

The results are presented in two sections. First, the GRAT-GUI can streamline the process to import and convert site meteorological data files into the ARL format before generating trajectory files. Figure 4 depicts the processed trajectory results of three meteorological stations using a standalone R script that read and plot the batch-processed trajectory files. For example, 2160 trajectory files were chosen, corresponding to the hourly meteorological data from each station from September 1, 2017 to September 30, 2017. The newly developed module can handle a large number of trajectories quickly. Depending on the available computational resources on the local machine, the GRAT-GUI can process significantly large meteorological data (*e.g.*, 10 years of hourly meteorological data) per GUI conversion in a noticeably short time. Specifically, when processing the aforementioned QA/QCed hourly meteorological data for one year, it takes about 4.5 min using a Windows 10 stand-alone laptop computer (Dell Precision 5530; Round Rock, TX, USA). The results show a major advancement compared to the current HYSPLIT model that only allows limited, *i.e.,* no more than six data entries, each time. A significant time saving is achieved using the developed GUI (Fig. 2) developed in this work.

Second, we conducted studies on stations at different sea levels and ran PSDF to detect potential sources and affected areas. Running PSDF requires 3 GB of available RAM using the newly developed GRAT-GUI, the majority of which is used to prepare the large data array and generate the high-resolution plot. For each selected chemical, the identified sources and influenced areas were visualized in a 2D plot with grid cells, which were embedded in a map. The plots directly showed the likelihood of being the potential source locations. Furthermore, the GRAT-GUI generated a set of CSV files and two-dimensional (2D) probability plots for each hazardous material and enables users to quantitively locate the influence area by the corresponding hazardous chemical.

Data processing times were recorded during the studies. The GRAT-GUI has been demonstrated to be an efficient solution for researchers to process large datasets without worrying about the bandwidth of the network and the security of data. In the test computer with 16 GB RAM, processing time for conversion of 5 min resolution data over a span of 9 years (2010 to 2019) took 5 minutes with GRAT GUI. In comparison, using the existing GUI of the HYSPLIT would take 1,000 h to complete. Therefore, this new GUI significantly reduces process time by allowing batch simulations. This tool offers advancement to users and the scientific community who are interested in rapid risk analysis. The last task tab is to compute trajectories. It provides the convenience and a quality user experience to set up the atmospheric model and compute trajectories without changing screens.

The outputs from each of the main tasks have been checked carefully. Results from the “Trajectory data processing” tab strictly follow the format from the established HYSPLIT model, and they can be modified to use other applications. Extensive data verifications have been performed by comparing against the results from the NOAA online HYSPLIT webpage using the same input data and credentials. The output results agree.

**Figure 5:**
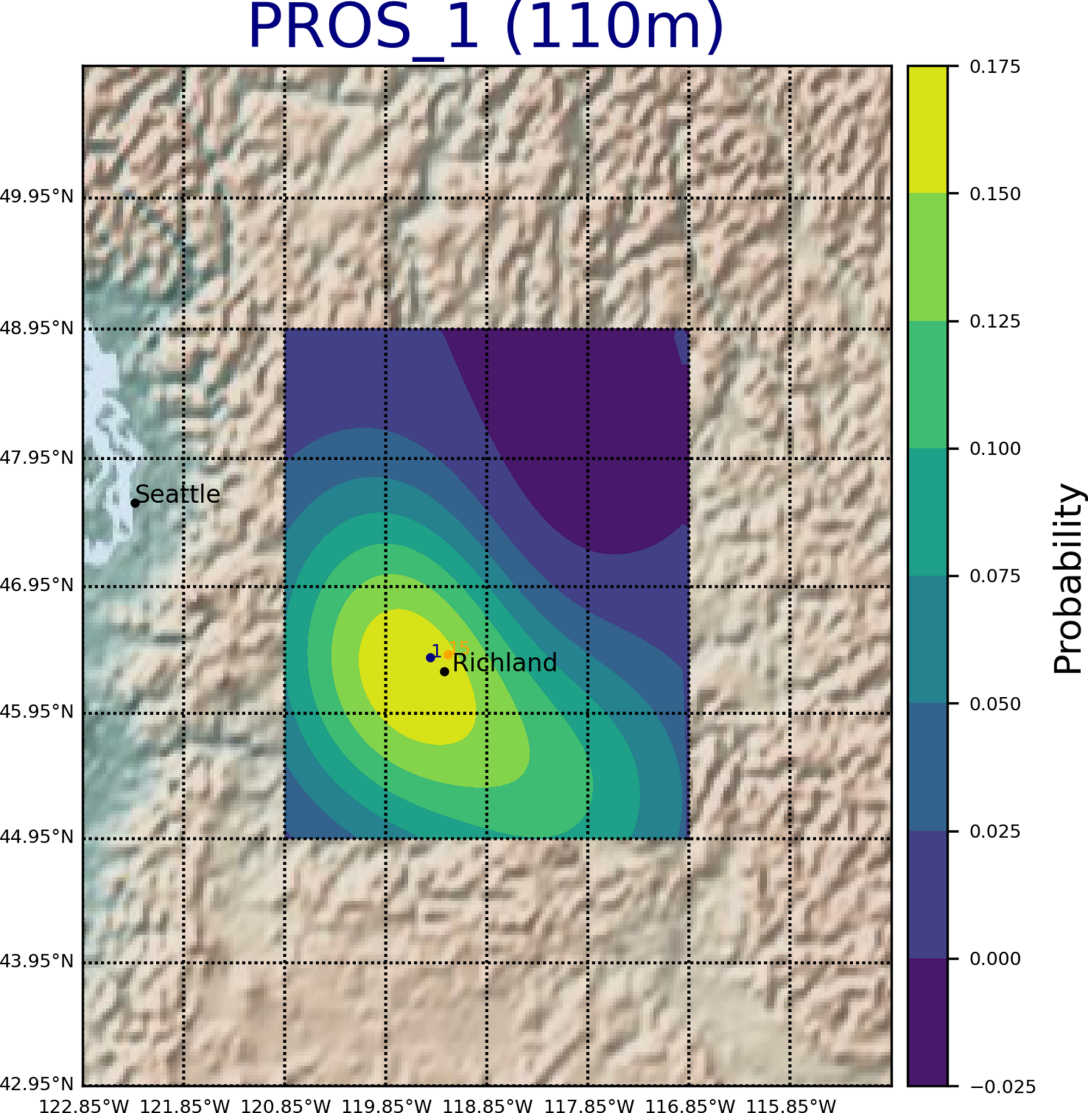
Study case of PSDF conducted at PROS station. The latitude and longitude of PROS are 46.39175309, −119.4117946, and the sea level is 110 m.

**Figure 6:**
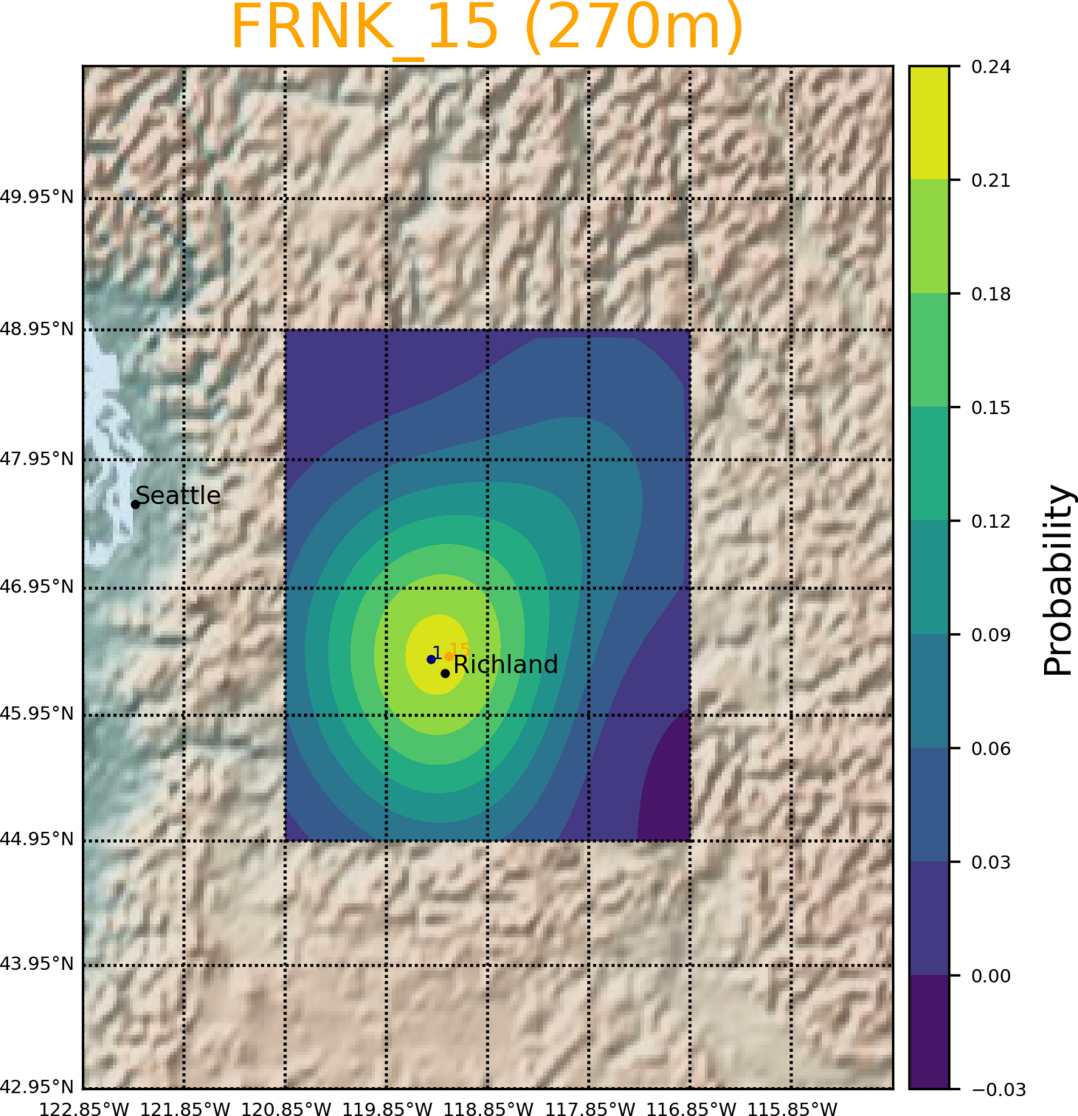
Study case of PSDF conducted at FRNK station. The latitude and longitude of FRNK are 46.4172515, −119.2378376, and the sea level is 270 m.

Figure 5 shows a representative PSDF result of a monitoring site at the sea level at Hanford. Similarly, Fig. 6 shows the PSDF result of a mountain site at Hanford. Because PDSF is a probability function, the results are portrayed as 2D probability maps (Pekney et al., 2006). Darker color corresponds to lower probability and lighter color higher probability. Both results show that the new software can offer rapid risk assessment of hazardous material release at a designated site.

The development of the GRAT-GUI provides a rapid tool to process a large amount of meteorological data based on physical models (namely algorithms within HYSPLIT) and advanced statistical functions like PSDF. Our results show a practical application following the DOE standards to perform risk analysis for different sites. Furthermore, this work offers a new capability of visualization of the source-receptor relationship of emission of hazardous materials. It is anticipated that this framework could lead to improvement of the safety analysis standard warranted for the safeguarding of the DOE facilities, personnel, and operation.

## Conclusions

It is critical to identify the potential emission sources and influenced areas due to hazardous chemical emissions in risk assessment. Back trajectories and advanced real-time VOC data from PTR-MS are used to compute PSDF and to determine the source–receptor relationship for a large data set. The NOAA well-established HYSPLIT model is used to compute trajectories, and it comes with HYSPLIT-GUIs for users to input their meteorological data.

The GRAT-GUI is created to speed up the analysis process by allowing a user to convert a large number of data entries of meteorological data and generate trajectory files within one set of operations. In this software development demonstration, 10 years of data are processed in less than 5 min, providing a huge time saving compared to the current GUI. Testing has confirmed that GRAT-GUI significantly improves the speed of the entire process to compute trajectories from users with big meteorological data. Studies from the sea-level monitoring station PROS and mountain station FRNK show that the PSDF results from GRAT-GUI can be used to identify the potential emission source and quantitively locate the influence area by using the PSDF statistical approach, and the overall GUI provides a rapid risk analysis module for geospatial risk analysis with diverse applications for the DOE sites and associated complex.

## Additional Information and Declarations

### Data Availability

The following information was supplied regarding data availability: The GRAT GUI is available at figshare: Hou, Hongfei; Ren, Huiying; Yu, Xiao-Ying; Royer, Patrick (2022): GRAT. figshare. Software. https://doi.org/10.6084/m9.figshare.19426742.v5. The data package is available under the “results\MetData” and “results\PTR-MS” subfolder of the installed GRAT-GUI. The code is available at Zenodo: jeffhonghou. (2022). jeffhonghou/GRAT: Initial release (V1.0.1). Zenodo. https://doi.org/10.5281/zenodo.7455345. The raw data is available at figshare: Hou, Hongfei; Ren, Huiying; Royer, Patrick; Yu, Xiao-Ying (2022): ARL Data from PROS station at Hanford site. figshare. Dataset. https://doi.org/10.6084/m9.figshare.19993964.v2. The DOE will also provide public access to these results of federally sponsored research in accordance with the DOE Public Access Plan (http://energy.gov/downloads/doe-public-access-plan).
